# A Case Study of Using Telehealth in a Rural Healthcare Facility to Expand Services and Protect the Health and Safety of Patients and Staff

**DOI:** 10.3390/healthcare9060736

**Published:** 2021-06-15

**Authors:** Jessica Anderson, Jitendra Singh

**Affiliations:** Minnesota State University Moorhead, Moorhead, MN 56563, USA; jessica.anderson@ihs.gov

**Keywords:** COVID-19, pandemics, telemedicine, leadership, health care, rural health

## Abstract

This case study aimed to gain an understanding of the implementation and usage of a telehealth program during the COVID-19 pandemic at a rural healthcare facility. An action research methodology, utilizing cycles of planning, implementation, review and adaptation was adopted to improve use of telehealth as COVID-19 cases continued to increase. Data was collected from literature review, examination of existing documents, review of gap and SWOT analysis, and examination of staffing plans. This helped in ensuring that adequate resources were in place to start and continue usage of telehealth. Additionally, review of the entire process was conducted as the program advanced through various phases of implementation. By conducting rigorous analysis and reflection, these data informed cycles of improvement in the telehealth program. Challenges surrounding the continuation and usage of telehealth have also been described. Because there is a paucity of research on the use of telehealth programs in rural healthcare facilities, especially during the pandemic, this study can provide practical tips to leaders and healthcare managers.

## 1. Introduction

Telehealth includes a broad array of services that facilitate delivery of medical consultation, patient education, health information services, and other related services via use of digital technologies. Technologies utilized in telehealth include but are not limited to video conferencing, health apps, mobile health, and other methods that promote patient monitoring from remote locations if needed. It is noteworthy that telehealth allows patients in rural settings to gain access to medical providers at larger facilities. These services can be used to address issues associated with physician shortages and access to specialty care in rural and remote locations [1]. A recent study suggested that 95% of patients who received medical consultation via telehealth were highly satisfied with the quality of care, ease of access, timeliness, and ability to integrate technology in their plan of care [2].

The COVID-19 Public Health Emergency was declared on 13 March 2020. For many healthcare facilities this meant limiting face-to-face services to protect the health and safety of patients, caregivers, and staff. According to the Center for Disease Control and Prevention [3], one major benefit of telehealth is maintaining continuity of care while avoiding the negative consequences of delayed preventive, chronic, or routine care. It has been reported that people living in rural areas are at increased risk of premature deaths due to chronic illness, stroke, and unintentional injury. Telehealth can serve as an effective method for providing care especially when medical professionals are able to monitor a patients’ condition, such as lung disease, from remote locations. This can lead to reductions in hospital admissions and even deaths in some cases [3]. Evidence suggests that there is a rise in the demand for telehealth services after COVID-19 hit. Compared to previous years, the usage in the month of October 2021 increased by 3.060% [4]. However, there is an increasing recognition that lack of adequate infrastructure, minimal or no training of health professionals, and restrictions on payment to many healthcare facilities for such services proved problematic. These barriers could lead to discontinuation of active telehealth programs. With the rise in COVID-19 cases around the world, it is important to examine new and on-going telehealth programs in rural facilities especially in areas where there are limited resources to address an outbreak. This action research project presents a case of a rural health facility located in northern Minnesota, where telehealth was implemented to provide care to patients during the pandemic. When the public health emergency was declared, the facility limited services to urgent and emergent care only and moved pharmacy dispensing outdoors. It was quickly realized that this model of care would not be sustainable for an extended period of time. Every discussion pointed to a telehealth program. Several factors surrounding implementation and continuation of the program have been discussed.

### Background and Rationale

Telemedicine refers to remote clinical services including clinical care, administrative services and ongoing/continuing medical education via use of technology. Telehealth can be defined as use of technology and electronic information to provide and enhance provision of health services, education, patient care, and health administration [5]. With an aging population, issues related to mobility and transportation can create obstacles for in-person hospital visits. Usage of telemedicine and advanced technologies including information processing, sensing, and artificial intelligence can help in providing support to individuals in their homes [6]. Additionally, timely access to information needed to make medical decisions, evidence based medicine and use of digital technologies and big data analytics can be help in managing health conditions of patients who suffer from two or more diseases and chronic health conditions [7].

Evidence suggests that telemedicine and telehealth programs can enhance patients’ access to care, help administrators manage scarce resources, support continuation in care and thereby reduce risk of transmission of coronavirus. Given rapid increases in the number of cases, many health centers have expanded telehealth visits. Approximately 95% of health centers reported that such visits were conducted during the pandemic [8]. A recent study conducted by Panicacci et al. (2021) presented a case of existing telemedicine system that was updated with new features to monitor and provide care to high risk COVID patients in their homes. This approach was extremely successful and led to a reduction in (or no) hospitalization, deaths, and positive feedback from patients and practitioners [9].

Utilizing telehealth during the pandemic can lead to long-term benefits for individuals who live in rural areas. This includes subspecialty services for patients who could not travel to urban locations to receive treatment. Research also suggests that usage of telemedicine enables effective management of care. Further, expansion of such services may have significant benefits for patients who seek mental health services. This in turn could benefit rural communities in cases such as deaths due to suicide, alcohol and drugs [10].

Data reported by the Center for Disease Control (CDC) and Health Resources and Services Administration (HRSA) clearly indicates a rapid decline in the usage of telehealth across health centers. For instance, during the week ending 26 June 2020, these visits declined to 35.8%. Further, a decrease of approximately 25% was reported when usage reports were examined in November 2020. It is important to note that health centers in the south and in rural areas reported the lowest usage of telehealth services over the period of 20 weeks when compared to urban areas. As COVID cases continue to increase, it is imperative to expand these services to limit exposure to the virus [8]. Telehealth programs can be successfully implemented in rural and remote locations, however, appropriate infrastructure issues such as reimbursement methods, access to internet, and licensure requirements still need attention [10]. Using action research methodology, this case study aimed to gain an understanding of the implementation and utilization of a telehealth program at a rural facility. By examining a variety of data sources, this project helps in building a greater understanding of the real world implementation of a telehealth program and challenges faced by leaders as they work on adoption of new processes/approaches in a rural health facility.

Due to the benefits associated with telehealth in rural locations, efforts must be made to examine implementation, usage, and reasons behind the increase in or declining use of telehealth in healthcare facilities. Dissemination of the findings from this research could help management and senior leaders as they work on implementing and/or expanding these services at their facilities.

## 2. Materials and Methods

This project was conducted using action research and action learning methods. Action learning is a method where individuals charged with real tasks work collaboratively to complete those activities in real conditions. Together, the entire team is exposed to challenges as they carry out real responsibilities. Once, an element of data collection and monitoring is added to the process, action research takes place. It is important to note that this monitoring aids in enhancing overall understanding of the process and generation of new knowledge, which is applicable in real life scenarios. Both action research and action learning build confidence in the new knowledge that is being generated and the outcomes of real world projects [11,12].

### 2.1. Study Site

This project was completed at White Earth Health Center (WEHC), an Indian Health Service (IHS) facility located in rural northern Minnesota in the United States of America. The facility provides ambulatory care services to approximately 10,000 American Indians and Alaska Natives annually. Indian Health Service (IHS), an agency within the Department of Health and Human Services, is responsible for providing health services to roughly 2.6 million American Indians and Alaska Natives who are part of 574 federally recognized Tribes [13].

### 2.2. Phases of Project/Research

This action research project was completed in three phases (highlighted below):

#### 2.2.1. Phase I—Strengths Weaknesses Opportunities Threats (SWOT) Analysis

Phase I of this project involved a thorough SWOT analysis by the organizational leaders. A SWOT analysis examines and evaluates internal strengths and weaknesses and external opportunities and threats in an organization’s environment. This analysis allows stakeholders to identify and understand means/assets, competencies and skill set, advantages the organization has, and how organizational leaders could utilize these resources to enhance competitiveness of its’ services. On the other hand, thorough analysis of external threats and opportunities allows organizational leaders to plan and expand their operations strategically based on market needs [14]. Organizational leaders utilized SWOT analysis to understand opportunities and strengths of telehealth as they worked with clinicians to prepare for challenges posed by rising COVID-19 cases. 

#### 2.2.2. Phase II—Gap Analysis and Creation of Staffing Plan and Implementation Plan

Organizational leaders conducted a gap analysis to assess the current state of telehealth utilization and how clinicians utilized telehealth to conduct patients’ visits. For the purpose of this project, gaps were defined as those occurrences in which WEHC resources, support mechanisms and procedures confirmed a difference when compared to the national evidence base [15]. This approach allowed WEHC to develop an efficient and integrated approach to delivering care that increases value for the patient population.

#### 2.2.3. Phase III—Ongoing Examination of Telemedicine Usage

To enhance participation and maintain the momentum for usage of telehealth services, it is extremely important to continuously examine the current process and develop change ideas that may be implemented in the care delivery process. This was also carried out at WEHC, and helped organizational leaders see the reasons behind declining usage when comparing the current state of usage against national trends seen throughout the US.

## 3. Results

### 3.1. Results of Phase I

**Figure 1:**
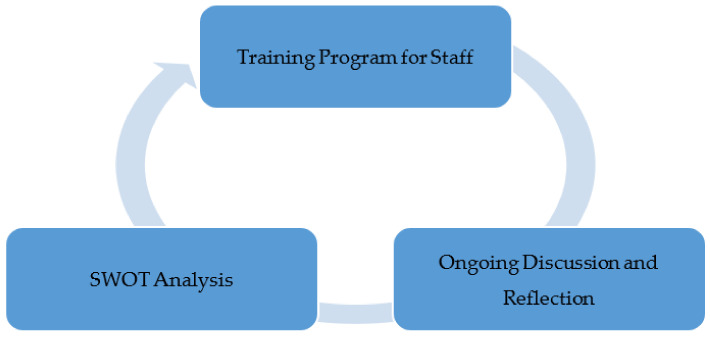
Phase I.

The SWOT analysis that follows was created to identify areas within the telehealth program that could be addressed to improve utilization. The literature has made it clear that the primary strength of telehealth is safety for both the patients and providers. A telehealth program would also allow for continuity of care and expanded services. There are also a number of opportunities to grow the program, however, weaknesses and threats can prevent the program from being successful (see Table 1; see Figure 1).

Reflection on these findings suggested that it was important to address lack of training and buy in from staff needed to conduct telemedicine visits. Telehealth can be a great mitigation strategy for COVID-19. However, more efforts were needed to direct staff to convert visits suitable for telehealth to telehealth and implement scheduled telework/telehealth on a regular basis (see Figure 1).

Once CMS issued 1135 waivers allowing IHS to collect revenue from telehealth visits a number of trainings were released to bring providers up to speed with offering the service. During this time frame, the medical staff were seeing very few patients so they were able to dedicate time to learning about telehealth services. The clinical applications coordinator built electronic health record (EHR) templates for the providers to use for their visits and also provided training on telehealth visit requirements. The physician lead for the telehealth program found a great continuing education piece from the American College of Physicians on incorporating telehealth into practice. Once the staff received more training on this piece, they were able to make clinical decisions without physically examining patients. Once the medical staff became more confident in the telehealth visits an audio visual component was incorporated using Cisco meeting. This service also required some staff training, demonstrations on use, and collaboration with nursing to connect patients to their provider through this service. The nursing staff played a vital role in sending patients connection information and directions to join Cisco meeting. They also made the initial call to patients for the meeting to perform screening prior to the provider joining the call.

Many of the staff were reluctant to provide telehealth on site as it seemed like more of an inconvenience for patients and themselves. When a staff member traveled internationally and had to quarantine upon return they provided telehealth from home. Their positive experiences providing telehealth from home created buy in with the other medical staff, bringing them on board with providing telehealth services. Having the option to work from home also gained provider buy in. The ability to work from home allowed many of the providers an alternate work schedule as they no longer had lengthy drives to the clinic.

Telehealth provided WEHC with opportunities to offer services in new ways. The CMS 1135 waivers allowed health care facilities to provide the service across state lines. This was beneficial because many of the consultative services WEHC patients received were located in North Dakota (ND). When patients needed services outside of what WEHC could offer they had to travel to urban locations in the state of ND. These patients were now able to receive these consultations for specialty services on site. This was essential to patients who needed cardiology or oncology consults, but were unable to easily get to urban locations.

Audio visual visits were the preferred method of telehealth for revenue generation purposes, but it was quickly realized that the internet connectivity on the reservation could not maintain connections for these visits. The CMS 1135 waivers allowed payment for services offered by phone, so telephone visits quickly became the preferred method of telehealth. To ensure medical staff privacy, google voice was used as opposed to using their actual phone numbers. If patients needed to share photos of something they could do so through text if they felt comfortable. If this method wasn’t reasonable the providers would either complete a home visit, or coordinate any in-person needs through the Tribal Home Health Nursing program.

The most significant threat to WEHC’s telehealth program is the potential discontinuation of the CMS 1135 waivers. The waivers are temporary and end no later than the termination of the pandemic, or 60 days from the date the waiver or modification is made unless the health and human services secretary extends it for periods of up to 60 days until the end of the pandemic [16]. By the end of May 2020, Medicare was reviewing their waivers and the data associated with them to consider making some of them permanent. The extension of expanded telehealth benefits under Medicare would please many providers, who have increased their use during the pandemic. Telehealth has provided another avenue for patients to receive medical services. While telehealth cannot replace in-person visits completely, it is a good alternative to meet patient needs. No longer offering the service could be detrimental to some patients and services.

### 3.2. Results of Phase II

**Figure 2:**
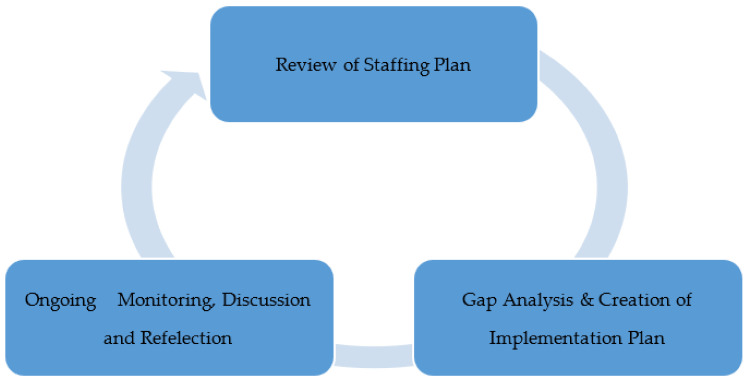
Phase II.

To better identify gaps in the current telehealth program, the team focused on two goals/objectives. Overall, the identified gap is that telehealth services were available and could be expanded, but they were not being utilized to the fullest extent possible (see Figure 2).

#### 3.2.1. Gap Analysis and Creation of Implementation Plan

This project intended to provide 25 percent of total primary care visits via telehealth by 1 November 2020. Several pieces of literature that were reviewed discussed how telehealth can provide access to care and continuity of care while keeping both the patients and healthcare providers safe. White Earth Health Center did not have a telehealth program prior to the pandemic due to limitations imposed by CMS. When the program was implemented at the end of March 2020, it slowly gained use. By early April, WEHC had developed and adopted a telehealth policy, developed and implemented EHR templates, and trained all medical staff on telehealth requirements. The telehealth program was launched. It slowly gained speed as it helped WEHC meet patient needs without coming to the clinic. By August, it was not uncommon to see 10 percent or less of primary care visits being completed using telehealth. The gap identified was the lack of telehealth visits taking place. The actions taken to close this gap were to direct staff to move visits that could be completed via telehealth to telehealth and develop a provider schedule that is more conducive to telehealth activity (see Table 2).

#### 3.2.2. Review of Staffing Plan

**Figure 3:**
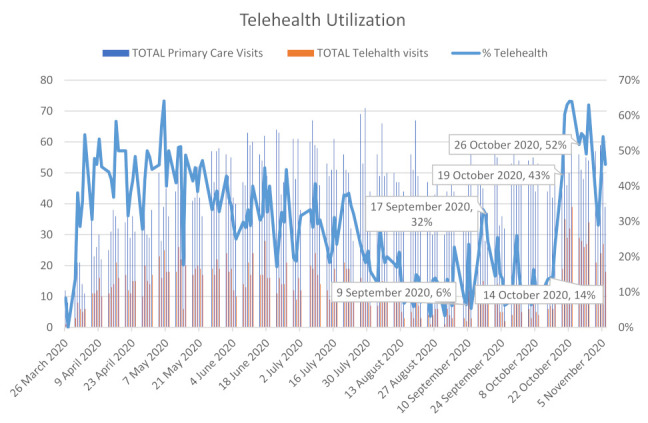
Telehealth Utilization.

Another primary objective of this project was to develop a schedule where 2–4 providers worked from home providing telehealth on a rotating basis. At the time, all providers were working on site, including those that provide telehealth. Some healthcare providers had moved to providing telehealth from home as a result of needing to quarantine [17]. While providers were in quarantine in mid-September, there was a notable increase in telehealth visits that had not been seen before (see Figure 3). This generated the idea of trialing a weekly rotating telework schedule to improve telehealth utilization. If the provider was not on site, there was no way for them to do anything other than telehealth. This also gave the healthcare facility the ability to place quarantined providers on telework if they were otherwise well.

### 3.3. Results of Phase III

**Figure 4:**
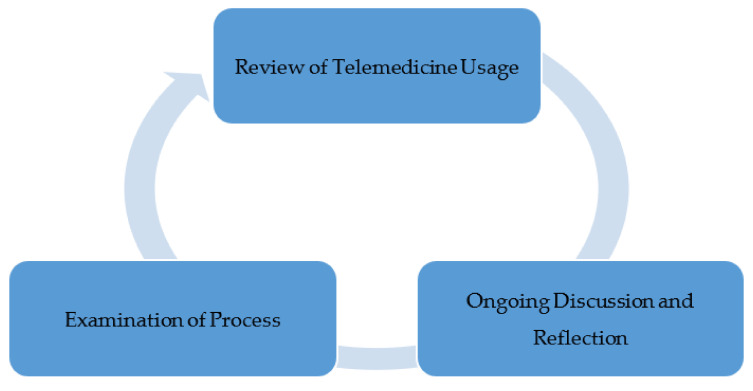
Phase III.

Ongoing review of medical visits indicated that in the early months of the pandemic, WEHC limited all services to urgent and emergent care only. The facility was seeing very few patients as people were afraid to come in unless they absolutely had to. Collections plummeted. WEHC did not have a telehealth program prior to the pandemic as Medicaid only paid IHS for face-to-face encounters. Medicaid is the largest portion of WEHC’s payer mix, so it was not fiscally responsible to offer a service WEHC could not be reimbursed for. IHS is prohibited from billing patients for services. Continuous review of literature and existing processes at WEHC revealed that when CMS announced the 1135 waivers, IHS was able to collect the face-to-face encounter rate by phone and other telehealth options, the race was on to rapidly implement a telehealth program. As the months passed, it was found that COVID was having very little impact on the area. There was feedback from the staff that many patients were becoming concerned about the lack of well-care taking place. Many patients were overdue for lab work, mammograms, and physicals. Area hospitals had resumed surgeries, which required pre-operative clearance for many WEHC patients. The limitations on services were decreased while continuing to offer telehealth as a primary option. By the end of summer, little to no telehealth visits were taking place (see Figure 3). This phenomenon also aligned with what was found during the literature review. There was a spike in telehealth in the spring and by fall the numbers decreased. Despite many discussions and other communications to staff, telehealth was not being used. WEHC leadership agreed that allowing the medical providers to telework would only give them the option to provide telehealth. This would also help maintain staff in the case there was a COVID outbreak in the clinic. The staff on telework would be well and able to come to work (see Figure 4). The telework schedule for medical staff was implemented during the week of 1 October 2020.

## 4. Discussion and Important Considerations

Prior to the COVID-19 Public Health Emergency, WEHC did not have a telehealth program. This was due to Medicaid restrictions that only allow IHS to bill for outpatient encounters when they are face-to-face visits. An encounter for an IHS facility, as defined by Minnesota Department of Health (MDH), is a face-to-face visit between a member eligible for Medical Assistance (MA) and any health professional at an IHS facility within a 24-h period ending at midnight. Since Medicaid comprises approximately 40 percent of WEHC’s third party payer mix, it was not fiscally responsible to limit collections by providing telehealth services regardless of reimbursement. WEHC receives only 27 percent of their funding through federal appropriations and the remainder of the funding comes by way of third party collections. All outpatient services at IHS facilities are at no cost to the patient. Effective 19 March 2020 for the Indian Health Service, telemedicine services, including telephonic, were included for the purpose of the face-to-face encounter payment methodology [17]. This meant that the Minnesota Department of Health (MDH) would reimburse IHS for telemedicine services of all types, which is vital to telehealth sustainability at WEHC [18]. 

The state of Minnesota was able to change their payment methodology due to the issuance of 1135 waivers. According to CMS (2017), when the president declares a disaster or emergency and the secretary of Health and Human Services (HHS) declares a public health emergency, the HHS secretary is authorized to temporarily waive or modify certain Medicare, Medicaid, and Children’s Health Insurance Program (CHIP) requirements to ensure sufficient health care items and services meet the needs of those affected by the emergency. Many of the waivers fall under section 1135 of the Social Security Act giving these waivers their name. CMS added equal coverage of audio only telehealth visits and 135 other allowable services as a result of these waivers. This more than doubled the number of services beneficiaries could receive via telehealth [19]. These flexibilities and allowances led to a surge in the number of beneficiaries getting telemedicine services. Before the public health emergency, approximately 13,000 beneficiaries received telemedicine in a week. As of April, nearly 1.7 million beneficiaries received telehealth services, and in total, over 9 million beneficiaries have received a telehealth service during the public health emergency [19]. Other changes made by Medicare include offering telehealth services to patients located in their homes and outside of designated rural areas, and reimbursement of telehealth visits in lieu of many in-person appointments. Furthermore, ability to communicate with patients across state lines, opportunity to see both new and established patients, and being able to conduct telephone visits helped in enhancing telemedicine services [20].

Payment for telehealth services was not the only barrier WEHC was facing. The facility is also in a very rural location and is considered an isolated hardship area due to its proximity to major hospitals. People who live in rural areas are more likely than urban residents to die prematurely from the five leading causes of death: heart disease, cancer, unintentional injury, chronic lower respiratory disease and stroke. Telehealth is just one approach to reduce barriers to care for those living rurally. Strategies that use cell phones have shown to be more helpful in providing services to these patients as more than 90 percent of rural residents own cell phones [3]. Unfortunately, telehealth options that rely on high-speed internet connections are not as helpful. Only about 60 percent of residents living in rural or Tribal areas have high-speed internet access, compared to over 95 percent of urban residents [3]. High-speed internet is commonly needed for audio video services.

Medicare beneficiaries represent a significant portion of the patient population at WEHC that experience disparities in digital access. More than 41 percent of Medicare patients lack access to a computer with high-speed internet connection at home, almost 41 percent do not have a smartphone with a wireless data plan, and more than 26 percent do not have access to either [21]. Evidence of telehealth un-readiness and inequities showed that those who were over 85, widowed, had a high school education or less, were Black or Hispanic, received Medicaid, or had a disability had even less digital access than other beneficiaries [21]. It was evident that federal telemedicine policy has focused on reimbursement and clinician ability to deliver care remotely and very little on disparities in digital access in order for patients to receive that care. The authors recommended expanding programs that provide reduced-cost phones or internet service to families with incomes 135 percent or more below the federal poverty level [21].

To summarize, WEHC quickly adopted a telehealth program near the end of March 2020 with utilization peaking by the end of April 2020. Due to the rural location of the health center, cases of COVID-19 remained low and telehealth usage declined starting in May and throughout the summer. An all-time low of 3% was experienced by the end of August with many patients returning on site for face-to-face visits. Literature from The Commonwealth Fund showed that the changes WEHC was experiencing were happening across the nation. By early April, in-person visits to ambulatory care practices had declined by nearly 60 percent. By mid-May, there was a rebound in the number of visits, but they were still about one-third lower than what was seen before the pandemic [22]. They also determined that as in-person visits dropped, telehealth visits increased rapidly before plateauing [22].

## 5. Facilitators and Challenges—Lessons for Healthcare Leaders

Leaders at WEHC adopted several strategies for increasing telehealth uptake. These include promoting and optimizing the use of telehealth services for safety purposes, communicating with payers to understand covered services, using tele-triage methods for assessing and caring for patients to decrease the number of people seeking in-person services, and providing outreach to patients with limited technology and connectivity. While WEHC was doing these things, there was still a decline in telehealth utilization. The medical staff were providing telehealth from the clinic, as opposed to their homes. As a result of healthcare staff needing to quarantine, WEHC noted an increase in telehealth visits. This led to the idea of incorporating regular telework into the staffing schedule to increase telehealth utilization. The section below highlights various facilitators and barriers that could help in adoption of telehealth programs.

### 5.1. Creating Buy-In

One of the key steps in implementing and continuing a telehealth program was to gain the buy-in of the medical staff early on for a rotating telework schedule to provide telehealth. Administrators in this project, as evident by documents, were able to develop and implement a 30 min telehealth appointment schedule and assign one provider to telework/telehealth on a rotating weekly basis. This grew to two providers as infection rates were on the rise. One objective was to accomplish the goal for two to four providers on telework at a time by hiring up to three contract physicians. This has been a difficult process compounded by COVID-19 and the northern Minnesota climate. Additional efforts for contracting physicians and clinical staff are needed to meet demands and increase utilization.

### 5.2. Communication

Communication with the team members, patients, and caregivers is important to enhance adoption of telehealth services. Similar to change initiatives, communication is central for ongoing usage of services. This communication needs to be between physicians, administrators, and amongst staff of various departments. Research suggests that if one fails to listen to others in an organization or in a different department, they will be limited in adoption of new practices [23,24]. This was illustrated in this study where open communication regarding telehealth programs helped in safe and effective patient care process. However, as time passed, the facility returned to usual in-person appointments for patients.

### 5.3. Financial Considerations

Financial considerations are extremely important so leaders and facilities can invest in additional telehealth equipment to facilitate connection with the specialists at specialty/subspecialty healthcare facilities located in urban areas. For the overall telehealth program, the CMS 1135 waiver that added equal coverage of audio only telehealth visits and subsequent adoption of the same by Minnesota Medicaid made it possible for WEHC to be reimbursed for nontraditional telehealth services. Had this change not been made, there would have been significant financial impacts to third party reimbursement for the organization. Inability to get reimbursed for audio only visits from both Medicaid and Medicare prior to the CMS 1135 waivers has proved detrimental for the success of telehealth programs. Due to poor internet connectivity in remote areas of the reservation, audio only visits became the most feasible form of telehealth. Before telehealth utilization started, WEHC was averaging about 10 primary care visits a day among 12 providers. Now, the facility has 60 to 70 primary care visits a day among 8 providers.

### 5.4. Evaluation

Leaders need to continuously monitor telehealth utilization rates to ensure there is on-going usage and no changes are needed. Change in utilization rate is a common evaluation method that was used for this project and will continue to be used in the future. For instance, key individuals leading the project communicated to staff about improving the utilization of telehealth. Daily monitoring of schedules allowed leaders to see if these visits were increasing or declining. When little to no change was noted a directive came from the top leadership that telehealth utilization must be a primary consideration. Ongoing monitoring and continuous communication helped in achieving the desired goal for the visits. Further, as next steps, leaders need to explore reasons behind low adoption of telehealth and then work on creating a plan to enhance participation in such programs.

### 5.5. Challenges for Leaders

There are significant concerns in the healthcare community about the state of telehealth once the pandemic ends. The 1135 waivers are only valid during the public health emergency. How do we take something away when the community has become used to having more options? There are already groups advocating to adopt the 1135 waivers moving forward. The value of telehealth must be communicated to the state, CMS, and Congress to ensure the 1135 waivers are adopted. Recruiting and maintaining the workforce is another big challenge in rural and tribal healthcare facilities. For instance, due to high turnover of physicians and clinical staff, administrators were faced with additional challenges in recruitment due to the COVID-19 pandemic. From April to December 2020, WEHC had three physicians and one nurse practitioner leave the organization for new positions. Failure to recruit and onboard staff led to problems as the telehealth program unfolded.

## 6. Limitations

This project presents a case study of a rural healthcare facility where a telehealth program was implemented during the COVID-19 pandemic. While several findings and lessons may still hold true, results may not be applicable to other healthcare facilities, especially in urban locations. Recommendations for future studies would be to attempt to include additional facilities and compare the findings with facilities in more urban locations.

## 7. Conclusions

COVID-19 has had a huge impact on rural facilities. While telehealth services will not substitute for every clinical visit to a doctor’s office, it is also important to note that these services have the possibility of being an important alternate. This project allowed us to gain an understanding of how a telehealth program was implemented and to gain insights into how usage of these services changed over a period of time. Operations changed significantly and at times they need to be adjusted daily. High turnover or retirement of essential staff since the start of the pandemic can put tremendous strain on the remaining staff. Recruiting new staff can be extremely difficult in rural facilities. Getting people to take interest in a rural area is challenging, but when candidates are from areas of milder weather that are less affected by COVID-19 it is hard to provide enough incentives to get them to join the team. Work in regard to telehealth will be ongoing. Efforts are needed to educate the community about what advantages telehealth can offer. These findings can help healthcare leaders as they plan on implementing such programs in their facilities.

## Figures and Tables

**Table 1:** SWOT Analysis.

Strengths	Weaknesses	Opportunities	Threats
Protect the health and safety of staff and patients	Patient and staff comfort with technology	Ability to offer consultativeservices data	The end of the public healthemergency and 1135 waivers thatallow IHS to offer this service
Maintain continuity of care	Lack of buy in from staff that will perform telehealth	Alternative clinicalschedulingmodels	Internet connectivity and speed invery rural low income areas
Expand services and remote monitoring services	Reliability of audio video technology used to complete telehealth visits		

**Table 2:** Gap Analysis.

Goals	Current State	Gap Identification	Efforts to Close the Gap
Provide 25 percentof total primarycare visits viaTelehealth by1 November 2020	Little to notelehealth takingplace despiteestablishing aprogram inMarch/April	Telehealthservices shouldbe used whenpossible for thesafety of patientsand staff	Direct staff to convertvisits suitable fortelehealth to telehealth and implement scheduledtelework/telehealth on a weekly rotating basis
Assign 2–4 providers to telework/telehealthon a weekly rotating basis	Providers are notProviding telehealthservices from home	Telehealth services should be used whenpossible for the safety of patients and staff	Create a schedule fortelework/telehealth on a weekly rotating basis
